# The Role of MicroRNAs in the Metastatic Process of High-Risk HPV-Induced Cancers

**DOI:** 10.3390/cancers10120493

**Published:** 2018-12-05

**Authors:** Joana M.O. Santos, Sara Peixoto da Silva, Natália R. Costa, Rui M. Gil da Costa, Rui Medeiros

**Affiliations:** 1 Molecular Oncology and Viral Pathology Group, IPO Porto Research Center (CI-IPOP), Portuguese Oncology Institute of Porto (IPO Porto), 4200-072 Porto, Portugal; joana.oliveira.santos@ipoporto.min-saude.pt (J.M.O.S.); peixotodasilva.sara@gmail.com (S.P.d.S.); maria.vieira.costa@ipoporto.min-saude.pt (N.R.C.); rmcosta@fe.up.pt (R.M.G.d.C.); 2 Faculty of Medicine of the University of Porto (FMUP), 4200-319 Porto, Portugal; 3 Research Department of the Portuguese League Against Cancer–Regional Nucleus of the North (Liga Portuguesa Contra o Cancro–Núcleo Regional do Norte), 4200-177 Porto, Portugal; 4 Center for the Research and Technology of Agro-Environmental and Biological Sciences (CITAB), University of Trás-os-Montes and Alto Douro (UTAD), 5001-911 Vila Real, Portugal; 5 Virology Service, Portuguese Oncology Institute of Porto (IPO Porto), 4200-072 Porto, Portugal; 6 Biomedical Research Center (CEBIMED), Faculty of Health Sciences of the Fernando Pessoa University, 4249-004 Porto, Portugal

**Keywords:** metastization, invasion, migration, microRNAs, high-risk HPV, cervical cancer, head and neck cancer, anogenital cancer

## Abstract

High-risk human papillomavirus (HPV)-driven cancers represent a major health concern worldwide. Despite the constant effort to develop and promote vaccination against HPVs, there is still a high percentage of non-vaccinated population. Furthermore, secondary prevention programs are not ubiquitous worldwide and not widely followed. Metastatic disease is the cause of the great majority of cancer-associated deaths, making it essential to determine its underlying mechanisms and to identify actionable anti-metastatic targets. Within certain types of cancer (e.g., head and neck), HPV-positive tumors show different dissemination patterns when compared with their HPV-negative counterparts, implicating HPV-related factors in the metastatic process. Among the many groups of biomolecules dysregulated by HPV, microRNAs have recently emerged as key regulators of carcinogenesis, able to control complex processes like cancer metastization. In this review, we present recent data on the role of microRNAs in the metastization of HPV-related cancers and on their possible clinical relevance as biomarkers of metastatic disease and/or as therapeutic targets.

## 1. Introduction

High-risk human papillomavirus (HPV) infection occurs at the basal cell layer of the stratified squamous epithelium (basal keratinocytes) and these viruses are established carcinogens of the cervix, head and neck, anus, penis, vagina and vulva [1]. Prophylactic vaccines against some high-risk and low-risk HPV types are available, but the populational coverage remains low [1]. High-risk HPVs, through the expression of E6, E7 and E5 oncoproteins, are able to immortalize the primary keratinocytes and induce genomic instability [1]. In fact, these oncoproteins regulate pathways that induce the hallmarks of cancer, including activation of invasion and metastization [1,2]. Indeed, studies showed that cells co-expressing the HPV16 oncoproteins E5, E6 and E7 have growth advantages, decreased adhesion and an increased migration and invasion, contributing to the development and metastization of HPV16-associated tumors [2,3,4].

Metastization is the process by which cancer spreads from a localized disease to a systemic disease [5]. The metastatic spread of the primary tumor is responsible for 90% of cancer-related deaths [6,7,8,9]. The metastization process of epithelial malignant neoplasia involves the acquisition of invasive potential by the primary tumor, through genetic and epigenetic alterations, followed by an expansive growth and invasion through the basement membrane [6,10,11]. Invasion of the basement membrane is promoted by decreased cell-cell and cell-matrix adhesion, through the alteration of the expression of adhesion molecules (e.g., E-cadherin, tight junctions) and enhanced cell motility [6,11]. Importantly, the expression of the HPV16 oncoproteins can promote the cellular invasive and migrative potential by the regulation of cadherins expression and associated pathways [3,4].

The vast majority of tumors can promote angiogenesis and/or lymphangiogenesis, since these events are pivotal points in tumor progression [6,10,12]. Neovessels not only deliver nutrients and oxygen for the tumor growth but also provide a route for tumor cells to enter into the circulation [12]. Intravasation requires the movement of cancer cells through the extracellular matrix and entry in lymph and/or blood vessels and/or serous cavities [6,8,10,13]. Circulating tumor cells (CTCs) face a significant challenge to survive in circulation until they extravasate at distant sites as disseminated tumor cells (DTCs) [6,8,10,14]. The pre-metastatic niche where DTCs are located is crucial for successful metastatic colonization [10,14,15]. Many of the tumor cells that extravasate may become dormant in these niches, extensively delaying the development of overt metastasis [8,10,16]. Despite the progress of research in this field, there is only a very limited success in decreasing the damaging impact of the metastasis in the clinical setting [6,7]. In fact, the treatment of metastatic disease and the discovery of reliable biomarkers to predict or detect early metastasis remain largely unmet challenges. 

MicroRNAs (miRNAs/miRs) are small non-coding RNAs with approximately 22 nucleotides in length that play a pivotal role in most essential biological events [17]. MiRNAs negatively modulate gene expression by post-transcriptional mechanisms, either by translational repression of the target mRNA or by induction of target mRNA cleavage [17,18,19]. Numerous miRNAs have been implicated in cancer initiation, progression and dissemination, either supporting or countering cancer development [18,20]. 

Importantly, the HPV oncoproteins can dysregulate the expression of several cellular miRNAs [19]. In vivo studies using HPV 16-transgenic mouse strains also support a role for multiple cellular miRNAs in HPV-related carcinogenesis [21,22,23,24]. HPV-encoded miRNAs are not widely accepted, although there are several studies suggesting their existence [25]. One of them is an in silico study by Gutiérrez et al. that reports the identification of 19 putative pre-miRNA candidates within HPV genomes [26]. Those viral miRNAs, according to their predicted target genes, could promote the development of cervical cancer, by participating in cellular longevity, cell cycle, apoptosis evasion, tissue invasion and metastization [26]. However, experimental data is needed for the validation of these results [26]. 

MiRNAs that are able to promote or inhibit metastization have been termed metastamiRs [27]. Furthermore, HPV oncoproteins can also dysregulate metastamiRs, and thus support the progression and metastization of HPV-induced cancers [19]. MiRNAs are relatively stable molecules and can be easily detected, being good candidates for cancer biomarkers and therapeutic targets [28]. This review focuses on the role of miRNAs in the biology of the metastatic process and their clinical usefulness in cancers associated with HPV. 

## 2. Cervical Cancer

Cervical cancer is the fourth most common cancer among women worldwide and a major cause of cancer-related mortality [29,30,31]. An association between the persistent infection with high-risk HPV and cervical cancer is firmly established and high-risk HPV is detectable in 99.7% of cervical cancers [31,32,33]. Several miRNAs have been recognized as contributors to migration, invasion and metastization in this type of cancer [34]. MiR-21 is upregulated by the HPV16 E7 oncoprotein, promoting cell proliferation and invasion [35]. Moreover, miR-21 is significantly overexpressed in invasive cervical carcinoma in comparison to carcinoma in situ, cervical intraepithelial neoplasia (CIN)-3, CIN-2, CIN-1 and normal cervix tissue [36]. Increased circulating miR-21 in cervical cancer patients is also associated with lymph node metastases and poor clinical stage [37]. In HeLa cells, miR-21 reduces RAS p21 protein activator 1 (*RASA1*) expression, promoting migration [37]. Rasa1 inactivates Ras from its active GTP-bound form to its inactive GDP-bound form. In agreement, HeLa cells transfected with miR-21 mimics showed increased phosphorylation of the Ras downstream targets protein kinase B (Akt) and extracellular signal–regulated kinases (Erk) [37]. Taken together, these studies suggest that miR-21 may play a pivotal role in metastization and that it could be a potential tool for cancer, restricting cell migration in cervical cancer [37].

The overexpression of miR-155 in cervical cancer tissue has also been correlated with lymph nodes metastases, as well as with International Federation of Gynecology and Obstetrics (FIGO) stage, vascular invasion and HPV status [38]. Moreover, patients with high miR-155 expression level had poorer overall survival compared with those with low miR-155 expression [38]. Increased miR-155 is an independent prognostic indicator for cervical cancer, which suggests that miR-155 could be a novel prognostic biomarker for these patients [38]. Nevertheless, a study performed in Caski cells showed that miR-155 overexpression decreased invasion capacities, inhibited cell proliferation and increased the chemosensitivity to cisplatin [39]. Furthermore, miR-155 inhibited epithelial-mesenchymal transition (EMT) induced by epidermal growth factor (EGF), through inhibition of mothers against decapentaplegic homolog 2 (*SMAD2*) and cyclin D1 (*CCND1*) and upregulation of tumor protein p53 (*TP53*) expression [39]. Thus, the influence of miR-155 in the metastization of cervical cancer remains controversial and needs further study.

MiR-218 is significantly reduced in cervical cancer tissue and its restoration inhibited cancer cell migration and invasion in both HPV-positive and HPV-negative cervical squamous cell carcinoma cell lines [40]. Additionally, laminin subunit beta 3 (*LAMB3*) which encodes a subunit of the protein Laminin-332, was identified as a direct target of miR-218 [40]. Other identified targets of miR-218 include scm-like with four MBT domains 1 (*SFMBT1)* and defective in cullin neddylation 1 domain containing 1 (*DCUN1D1*) [41]. Overexpression of *SFMBT1* induced EMT, migration and invasion and *DCUN1D1* also induced migration and invasion [41]. Importantly, HPV16 E6 may be responsible for the decrease of miR-218 expression in cervical cancer cells, but it remains to be demonstrated whether this is a p53-dependent or independent phenomenon [19,41]. Recently, in vitro studies showed that *DCUN1D1* is also a target gene of miR-195 and this miRNA is associated with reduced proliferation, migration and invasion of cervical cancer cell lines [42]. Moreover, reduced expression of miR-195 was associated with lymph node metastases and an advanced clinical stage in cervical cancer patients [42]. Interestingly, miR-195 expression is reduced by HPV16 E6 oncoprotein [42].

Downregulation of miR-375 in cervical cancer tissues is also correlated with pelvic lymph node metastases, FIGO stage, and other indicators of poor prognosis [43]. Suppression of miR-375 increases the expression of specificity protein 1 (*SP1*) and consequently promotes cell proliferation, migration and invasion in vitro [43]. Downregulation of miR-375 may occur due to the hypermethylation of its promoter, which is mediated by HPV16 E6 through DNA methyltransferase 1 (DNMT1) [19,44]. Furthermore, miR-375 overexpression can suppress EMT in cervical cancer cells by downregulating metastases-associated lung adenocarcinoma transcript 1 (MALAT1) [44]. MALAT1 is a long non-coding RNA (lncRNA) upregulated in cervical cancer whose knockdown significantly reduces cell growth rate and invasion and increased cell apoptosis and the expression of miR-124 [45]. Growth factor receptor bound protein 2 (*GRB2*) was identified as a target of miR-124 and its knockdown significantly reduced cell invasion and increased apoptosis [45]. Therefore, MALAT1 has been suggested to promote tumor development by “spongeing” miR-124, which leads to an upregulation of *GRB2* and consequently to increased cell invasion [45].

MiR-34a and miR-23b seem to be downregulated by HPV E6 indirectly, since their expression is driven by p53 [19]. Downregulation of miR-23b may also be caused by promoter hypermethylation of its host gene by DNMT1 [19]. Importantly, forced expression of miR-34a repressed invasiveness of HeLa cells by inhibition of the Notch pathway and consequent decreased urokinase plasminogen activator (*uPA*) expression [46]. Moreover, increased expression of *uPA* can also be promoted by miR-23b repression, resulting in increased migration of cervical cancer cell lines [47].

Plexin B1 (*PLXNB1*) is upregulated in cervical cancer tissue and HeLa cells, promoting cell proliferation, migration and invasion [48]. *PLXNB1* upregulation may be caused by the downregulation of miR-214, since this miRNA was found to have a binding site within the 3’- untranslated region (UTR) of *PLXNB1* [48]. Ectopic expression of miR-214 also inhibited the proliferation, migration and invasion ability of HeLa cells [48]. Another study also showed that miR-214 is downregulated in cervical cancer tissue, and polypeptide N-acetylgalactosaminyltransferase 7 (*GALNT7*) was also identified as a target of this miRNA [49]. The knockdown of *GALNT7* in cervical cancer cell lines inhibited cell proliferation, migration, and invasion [49].

MiR-205 expression is frequently increased in human cervical cancer, promoting cell proliferation and migration [50]. Both cysteine rich angiogenic inducer 61 (*CYR61*) and connective tissue growth factor (*CTGF*) were identified as potential targets of miR-205 and may play important roles in cervical carcinogenesis [50].

Upregulation of miR-133b accompanies cervical carcinoma progression [51]. In vitro studies showed that miR-133b enhances cell proliferation and colony formation by targeting the mammalian sterile 20-like kinase 2 (*MST2*), cell division control protein 42 homolog (*CDC42*) and ras homolog gene family member A (*RHOA*) and consequently phosphorylating Akt1 and Erk [51]. Mice transplanted with CaSki cells overexpressing miR-133b had a significant increase in the number of lung metastatic foci when compared with the control group [51].

MiR-125a is downregulated in cervical cancer patients, and negatively correlated with tumor size, FIGO stage, and preoperative metastases [52]. Moreover, miR-125a downregulation may be due to p53 inactivation by HPV E6 [52]. Overexpression of miR-125a significantly suppressed the growth, invasion and EMT in both in vitro and in vivo studies by targeting and reducing the expression of signal transducer and activator of transcription 3 (*STAT3*) [52]. Thus, miR-125a may be a potential therapeutic target for cervical cancer [52].

Increased expression of miR-20b is also promoted by HPV E6 [53]. In vitro studies showed that overexpression of miR-20b leads to a decrease in TIMP metallopeptidase inhibitor 2 (*TIMP2*) and thus induces cell morphological alterations and EMT, promoting migration and invasion [53].

It was recently reported that the HPV E6 and E7 oncoproteins enhance the expression of c-Myc, which in turn mediates the downregulation of miR-146a-5p [54]. In cervical cancer and keratinocyte cell lines, overexpression of miR-146a-5p inhibited proliferation and migration by targeting lysine-specific demethylase 2B (*KDM2B*) [54].

HPV16 E7 also promotes miR-27b expression in cervical cancer cell lines by increasing DiGeorge syndrome critical region gene 8 (DGCR8), which in turn decreases the expression of miR-27b target polo like kinase 2 (*PLK2*) and promotes proliferation and invasion [55]. Another study also identified peroxisome proliferator activated receptor gamma (*PPARG*) as a miR-27b target and its downregulation increases the expression of sodium-hydrogen exchanger isoform 1 (NHE1), inducing proliferation and invasion [56].

The expression of miR-106b-5p is upregulated in cervical cancer and significantly correlated with the number of metastatic lymph nodes [57]. In silico analysis demonstrated that miR-106b-5p promotes the progression of cervical cancer by modulating the expression of glycogen synthase kinase 3 beta (*GSK3B*), vascular endothelial growth factor A (*VEGFA*), and protein tyrosine kinase 2 (*PTK2*) genes, which are genes that play a crucial role in phosphatidylinositol 3-kinase (PI3K)-Akt signaling and focal adhesion [57].

## 3. Other Anogenital Cancers

Besides cervical cancer, HPV infection has also been etiologically associated and implicated with the development of other anogenital cancers (vulva, vagina, anus and penis) [58,59,60]. An estimated 85% of anal cancers and approximately a half of the vulvar, vaginal and penile cancers are attributable to HPV [61,62]. However, apart from the many studies that associated HPV with anogenital cancers, little is known about how HPV actually contributes to the development of these cancers [61].

Vulvar cancer is rare, representing about 5% to 6% off all gynecological malignancies [60,63,64]. Globally, HPV is a major player in vulvar cancer, contributing to a quarter of the invasive vulvar cancers and a large part of vulvar intraepithelial lesions [63]. The incidence of vulvar cancer has been increasing over the last decades, particularly in younger women, less than 50 years old [65,66]. In vulvar cancer, the dysregulation of miRNAs has remained a largely uncharted territory, with only slow progress being made in the last years [67]. The association of a few miRNAs with invasion and metastases in vulvar cancer was assessed in HPV-negative samples [68,69,70] or in samples where HPV infection was not confirmed [71,72]. Only de Melo Maia et al. (2013) made a miRNA portrait in vulvar cancers which differentiates HPV-positive and HPV-negative samples [67]. In this study, 25 miRNAs were differentially expressed in HPV-positive and HPV-negative tumors. While 10 miRNAs were upregulated, 15 were downregulated in HPV-positive tumors [67]. Among those miRNAs dysregulated in HPV-positive tumors, only miR-16 and miR-19b-1 have been previously associated with cell invasion in ovarian and cervical cancers but their role in HPV-related vulvar cancer remains to be clarified [67,73,74].

Vaginal cancer is also considered a rare malignancy, accounting for about 2% of all gynecologic cancers [60]. Several risk factors have been described for vaginal cancer, like smoking, immunosuppression and high-risk sexual behavior [60]. In addition, HPV also plays an important etiological role in vaginal cancers [60,75,76], of which 64% are HPV-positive [75,77,78]. Probably due to its rarity, no studies have addressed miRNA dysregulation in vaginal cancer.

With an estimated 27,000 new cases worldwide per year, anal cancer is considered relatively uncommon in the general population [79,80]. Nevertheless, the incidence of anal cancer is increasing [81] and high-risk HPV is considered responsible for 85% of the cases [61,62,80,82]. Therefore, the increasing incidence of this malignancy has been associated with a rise in prevalence of factors associated with the incidence and persistence of anal HPV infection [81]. The existing data concerning miRNAs in anal cancer is extremely scarce [83] and no miRNAs have been specifically associated with the metastatic process.

Penile cancer is another rare disease [84,85], occurring predominantly in elderly men (50–70 years) and in men with low socioeconomic status, and is associated with a high morbidity and mortality [60,85,86,87]. Squamous cell carcinoma is the most frequent histological type and some well-established risk factors include poor hygiene, non-circumcision, phimosis, and HPV infection [60,88]. Almost 30% to 40% of all penile cancers are related to HPV, and HPV infection is related to specific histological subtypes [82,84,89,90,91,92]. Several studies have described miRNA dysregulation in penile cancer [93,94,95,96], but no association was found with metastization in HPV-related penile cancer. In a study by Barzon et al., miR-218 expression was found to be downregulated in high-risk HPV-positive penile squamous cell carcinomas when compared to HPV-negative samples [94]. Additionally, Peta et al. showed that HPV E6 can upregulate the epidermal growth factor receptor (*EGFR*) via miR-146a downregulation in penile cancer [95]. The results of Barzon et al. and Peta et al. are in agreement with findings about these miRNAs for cervical cancer. Results from other teams working with different types of HPV-related cancer support the hypothesis that these miRNAs play a role in cancer invasion and metastization [40,41,54,97,98,99], but whether they play a similar role in penile cancer remains to be confirmed.

Scrotal squamous cell carcinoma has also been associated with HPV infection [100]. High-risk HPV infection (HPV18 and HPV16) has been proposed to be involved in the pathogenesis of this very rare cancer (1.5 per 1 million person-years in Western countries) [88,100,101]. To date, there are no data concerning the expression of miRNAs in this malignancy.

## 4. Head and Neck Cancers

Head and neck cancers (HNC) are the fifth most common cancers in the world, with high rates of mortality [102]. There are several risk factors associated with this malignancy, including high-risk HPV infection [103]. HNC can arise from the oral and nasal cavity, larynx, hypopharynx, and oropharynx [103]. From these locations, only three have been associated with high-risk HPV infection, namely oropharynx, oral cavity and larynx [104]. The incidence of HPV-related HNC has increased significantly in the last decade [103].

As for cervical cancer, several metastamiRs have been identified in HPV-positive HNC. Specifically, a set of miRNAs, namely miR-151, miR-152, miR-324-5p, miR-361 and miR-492 is significantly associated with distant metastases in HPV-derived oropharyngeal carcinoma [105].

MiR-363 is also upregulated in HPV16-positive HNC compared with HPV-negative cases [106]. Myosin 1B (*MYO1B*) is targeted by miR-363, and siRNA knockdown of *MYO1B* expression reduces migration in cell lines of HNC [106]. Thus, overexpression of miR-363 reduces cellular migration in HPV-positive HNC [106].

Transfection of HPV-positive or negative HNC stem cell lines with miR-34a mimics downregulates the expression of transcription factors such as Snail and Twist that regulate stem cell properties and EMT, suggesting that miR-34a may reduce cancer cell “stemness” and EMT, thereby reducing the invasions capacities of these cell lines, regardless of their HPV status [107]. Kumar et al. also demonstrated that miR-34a is downregulated in both HPV-positive and HPV-negative HNC tissue and cell lines [108]. In this study, miR-34a expression inhibited tumor cell proliferation and colony formation by downregulating E2F transcription factor 3 (*E2F3*) and survivin [108]. Additionally, miR-34a transfection inhibited cell migration and tumor angiogenesis by downregulating VEGFA [108].

The expression of miR-20a was also found to be significantly elevated in oral HNC [109], and the silencing of HPV16 E7 in Cal27 cells induced miR-20a downregulation [109]. Paradoxically, restoring HPV16 E7 expression and consequently miR-20a expression inhibited Cal27 cell proliferation, migration and invasion [109]. These results are in contrast with others showing that HPV oncoproteins increase the migration and invasive potential of cells and deserve further confirmation, as does the role of miR-20a [3,4].

Sannigrahi et al. reported that miR-139-3p overexpression downregulates HPV16 mRNA/proteins and consequently decreases cell proliferation and migration [110]. In fact, HPV-positive HNC showed low miR-139-3p expression, which may be due to promoter hypermethylation of phosphodiesterase 2A (*PDE2A*), the gene harboring miR-139-3p [110].

Overall, dysregulated expression of miRNAs is associated with tumor cell migration, invasion and metastization in HNC, but most studies do not report HPV status, thereby limiting the conclusions that may be drawn concerning the relationship between HPV and miRNAs in this setting. Additional studies on this topic are required, given that HPV is clearly able to dysregulate the expression of several critical miRNAs and influence the prognosis of HNC patients [19,111].

## 5. Other Cancers Potentially Related to High-Risk HPV

High-risk HPVs have been found in cancers from several other locations than cervix, other anogenital and head and neck [112,113]. Several studies show a possible association between HPV and other malignancies, such as esophageal and lung cancers [112,114,115,116], although the role of HPV in these settings remains controversial.

### 5.1. Esophageal Cancer

Esophageal cancer, in particular esophageal squamous cell carcinoma (ESCC), has long been proposed to be associated with HPV [112,117,118,119]. Interestingly, Cui et al. observed that miR-203 showed heavy CpG methylation in HPV16-positive ESCCs compared to HPV-negative cancers [120]. The consequently lower expression of miR-203 in HPV16-positive cases was significantly associated with the development of lymph node metastases [120]. The authors also suggested that miR-203 hypermethylation could be a potential biomarker for HPV16-positive ESCC and that targeted delivery of this miRNA may serve as a preventive or therapeutic approach for ESSC [120]. The impact of metastamiRs in esophageal cancer remains largely to be determined.

### 5.2. Lung Cancer

The involvement of HPV in lung cancer is still a matter for discussion [114,115,116,121]. Interestingly, HPV16 oncoproteins seem to contribute to non-small cell lung cancer (NSCLC) progression by promoting hypoxia-inducible factor 1-alpha (HIF-1α)/VEGF-mediated tumor angiogenesis [122] and by enhancing EMT through the activation of PI3K/Akt/HIF-1α signaling [123]. A study by Wu et al. reported that HPV16 E6 may upregulate metastasis-associated protein-1 (*MTA-1*) gene expression through inhibition of miR-30c-2* in NSCLC tissues. The same study correlated miR-30c-2* and MTA-1 expression with tumor stage and lymph node metastases [124]. High expression of MTA-1 was also associated with a poor clinical outcome, tumor recurrence and a poor therapeutic response. Therefore, the expression levels of this miRNA and MTA-1 could potentially function as prognostic biomarkers for patient outcome, recurrence and therapeutic response of patients with lung cancer. The same authors also pointed out that the possible signaling pathway HPV16/miR-30c-2*/MTA-1 might offer a new therapeutic target for patients with HPV-associated lung cancer [124].

## 6. Conclusions

**Figure 1:**
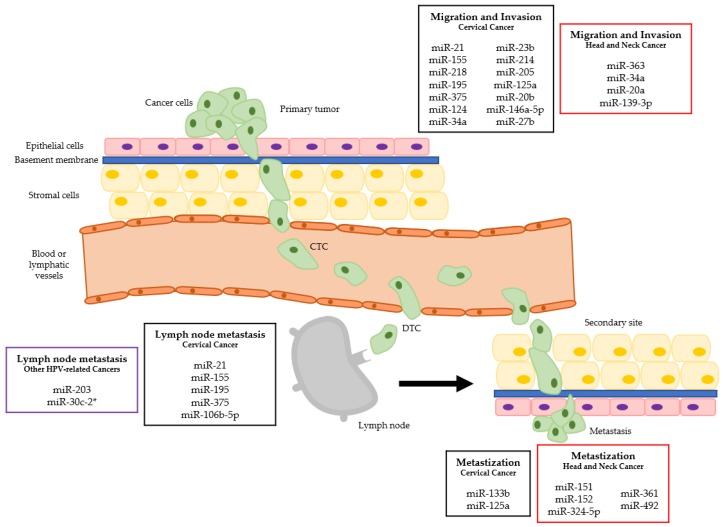
MiRNAs dysregulation in the different metastatic steps of HPV-related cancers. (CTC—circulating tumor cell; DTC—disseminated tumor cell; squares in black refer to cervical cancer, in red to head and neck cancer and in purple to other HPV-related cancers).

High-risk HPVs are oncogenic viruses associated with several malignancies, most prominently cervical cancer. In this review, we provide an overview of the metastamiRs found in HPV-driven cancers (Table 1, Table 2 and Table 3) and their relation with the different steps of the metastization process (Figure 1). Some of those miRNAs hold promise as prognostic biomarkers and as potential targets for novel anti-cancer therapies.

There is a great lack of information about metastamiRs on less-frequent tumors, like the ones in vulva, vagina, anus and penis. Additionally, a great number of studies concerning miRNAs and metastization in cancers possibly related to HPV do not take into consideration the tumor HPV status. Further directions in this field clearly include the study of less-frequent cancers and the determination of common patterns of HPV-related miRNA dysregulation, as well as the validation of selected miRNAs for clinical applications.

## Figures and Tables

**Table 1:** MetastamiRs in cervical cancer.

Cancer	MiRNA	Expression	Type of Samples	Targets	Biological Significance
**CERVICAL CANCER**	miR-21	Upregulated [35,36,37]	Serum [37] Tissue [36] HeLa cell line [35,36,37] SiHa cell line [36] CaSki cell line [36]	*RASA1* [37]	Promoted cell migration and invasion [35,37] Associated with lymph node metastases [37]
	miR-155	Upregulated [38]	Tissue [38] HeLa cell line [38]	---	Correlated with lymph nodes metastases and vascular invasion [38] Promoted cell migration and invasion [38]
		Upregulated [39]	CaSki cell line [39]	*SMAD2* [39] *CCND1* [39]	Decreased proliferation [39] Inhibited EGF-induced EMT, migration and invasion [39] Increased chemo-sensitivity [39]
	miR-218	Downregulated [40,41]	Tissue [40,41] CaSki cell line [40] ME180 cell line [40] SiHa cell line [41] HeLa cell line [40,41]	*LAMB3* [40] *SFMBT1* [41] *DCUN1D1* [41]	Associated with cell migration and invasion [40,41] Induction of EMT and invasion [41]
	miR-195	Downregulated [42]	Tissue [42] HeLa cell line [42] SiHa cell line [42]	*DCUN1D1* [42]	Associated with lymph node metastases [42] Mediated cell proliferation, migration and invasion [42]
	miR-375	Downregulated [43,44]	Tissue [43] SiHa cell line [43,44] CaSki cell line [43,44]	*SP1* [43] LncRNA MALAT1 [44]	Correlated with pelvic lymph node metastases [43] Promoted cell proliferation, migration and invasion [43] Modulation of EMT [44]
	miR-124	Downregulated [45]	Tissue [45] HeLa cell line [45] CaSki cell line [45] SiHa cell line [45]	*GRB2* [45]	Increased cell invasion [45]
	miR-34a	Transfection with pre-miR-34a [46]	HeLa cell line [46]	*NOTCH1* [46] *JAGGED1* [46]	Repression of invasion [46]
	miR-23b	Downregulated [47]	SiHa cell line [47] CaSki cell line [47]	*uPA* [47]	Increased cell migration [47]
	miR-214	Downregulated [48]	Tissue [48] HeLa cell line [48]	*PLXNB1* [48]	Promoted cell proliferation, migration and invasion [48]
	miR-205	Upregulated [50]	Tissue [50] HeLa cell line [50] CaSki cell line [50]	*CYR61* [50] *CTGF* [50]	Promoted cell proliferation and migration [50]
	miR-133b	Upregulated [51]	Tissue [51] CaSki cell line [51] SiHa cell line [51] Female SCID mice [51]	*MST2* [51] *CDC42* [51] *RHOA* [51]	Enhanced cell proliferation and colony formation [51] In mice increased the number of lung metastatic foci [51]
	miR-125a	Downregulated [52]	Tissue [52] SiHa cell line [52] HeLa cell line [52]	*STAT3* [52]	Correlated with preoperative metastases [52] Modulation of EMT [52]
	miR-20b	Upregulated [53]	Tissue [53] HeLa cell line [53] SiHa cell line [53] CaSki cell line [53]	*TIMP2* [53]	Induced EMT, migration and invasion [53]
	miR-146a-5p	Downregulated [54]	Primary HFKs cell line [54] HeLa cell line [54] SiHa cell line [54] CaSki cell line [54]	*KDM2B* [54]	Promoted cell proliferation and migration [54]
	miR-27b	Upregulated [55,56]	Tissue [55,56] CaSki cell line [55,56] SiHa cell line [55,56]	*PPARG* [56] *PLK2* [55]	Promoted cell proliferation and invasion [55,56]
	miR-106b-5p	Upregulated [57]	In silico studies [57]	*GSK3B* [57] *VEGFA* [57] *PTK2* [57]	Correlated with the number of metastatic lymph nodes [57]

**Table 2:** MetastamiRs in HPV-positive head and neck cancer.

Cancer	MiRNA	Expression	Type of Samples	Targets	Biological Significance
**HEAD AND NECK CANCER (HPV-POSITIVE)**	miR-151	Upregulated [105]	Tissue (oropharyngeal cancer biopsies) [105]	-	Associated with distant metastases [105]
	miR-152	Downregulated [105]	Tissue (oropharyngeal cancer biopsies) [105]	-	Associated with distant metastases [105]
	miR-324-5p	Upregulated [105]	Tissue (oropharyngeal cancer biopsies) [105]	-	Associated with distant metastases [105]
	miR-361	Upregulated [105]	Tissue (oropharyngeal cancer biopsies) [105]	-	Associated with distant metastases [105]
	miR-492	Downregulated [105]	Tissue (oropharyngeal cancer biopsies) [105]	-	Associated with distant metastases [105]
	miR-363	Upregulated [106]	Tissue (head and neck squamous cell carcinoma) [106]	*MYO1B* [106]	Reduced cell migration [106]
	miR-34a	Transfection of mir-34a mimics [107]	Spheroid head and neck cancer cell lines [107]	-	Reduced invasion capacity [107]
		Downregulated [108]	Tissues (head and neck squamous cell carcinoma) [108] UM-SCC-74A cell line [108] UM-SCC-74B cell line [108] SCID mice [108]	*E2F3* [108]	Promotes cell proliferation, migration and angiogenesis
	miR-20a	Upregulated [109]	Tissue (oral squamous cell carcinoma) [109] Cal27 cell line [109]	-	Inhibited cell proliferation, migration and invasion [109]
	miR-139-3p	Downregulated [110]	Head and neck cancer tissues [110] UPCI-SCC-090 cell line [110]	*HPV16-E1* [110]	Induced cell proliferation and migration [110]

**Table 3:** MetastamiRs in other cancers potentially related to high-risk HPV.

Cancer	MiRNA	Expression	Type of samples	Targets	Biological Significance
**OTHER HPV-RELATEDCANCERS**	miR-203	Downregulated [120]	Tissue (Esophageal squamous cell carcinoma) [120]	-	Promoted lymph node metastases [120]
	miR-30c-2*	Downregulated [124]	Tissue (NSCLC) and TL1 cell line [124]	*MTA-1* [124]	Correlated with tumor stage and lymph node metastases [124]
